# Test–retest reproducibility of a multi‐atlas automated segmentation tool on multimodality brain MRI

**DOI:** 10.1002/brb3.1363

**Published:** 2019-09-04

**Authors:** Thiago J. R. Rezende, Brunno M. Campos, Johnny Hsu, Yue Li, Can Ceritoglu, Kwame Kutten, Marcondes C. França Junior, Susumu Mori, Michael I. Miller, Andreia V. Faria

**Affiliations:** ^1^ Department of Neurology University of Campinas Campinas Brazil; ^2^ Department of Radiology The Johns Hopkins University School of Medicine Baltimore Maryland; ^3^ AnatomyWorks LLC Baltimore Maryland; ^4^ Department of Biomedical Engineering The Johns Hopkins University Baltimore Maryland

**Keywords:** automated segmentation, multimodality brain MRI, reproducibility, test–retest

## Abstract

**Introduction:**

The increasing use of large sample sizes for population and personalized medicine requires high‐throughput tools for imaging processing that can handle large amounts of data with diverse image modalities, perform a biologically meaningful information reduction, and result in comprehensive quantification. Exploring the reproducibility of these tools reveals the specific strengths and weaknesses that heavily influence the interpretation of results, contributing to transparence in science.

**Methods:**

We tested–retested the reproducibility of MRICloud, a free automated method for whole‐brain, multimodal MRI segmentation and quantification, on two public, independent datasets of healthy adults.

**Results:**

The reproducibility was extremely high for T1‐volumetric analysis, high for diffusion tensor images (DTI) (however, regionally variable), and low for resting‐state fMRI.

**Conclusion:**

In general, the reproducibility of the different modalities was slightly superior to that of widely used software. This analysis serves as a normative reference for planning samples and for the interpretation of structure‐based MRI studies.

## Graphical abstract

Addressing the methodological reproducibility of imaging postprocessing is essential for planning and data interpretation. MRICloud showed reproducible results for whole‐brain, multimodal, structure‐based quantification. Structural analyses (volumetric and DTI) show higher reproducibility than rsfMRI analysis.

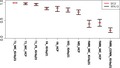

## 1 HIGHLIGHTS

- Addressing the methodological reproducibility of imaging postprocessing is essential for planning and data interpretation.
- MRICloud showed reproducible results for whole‐brain, multimodal, structure‐based quantification.
- Structural analyses (volumetric and diffusion tensor images) show higher reproducibility than rsfMRI analysis.

## 2 INTRODUCTION

Integrative analysis of multiple MRI contrasts is increasingly popular because the power to discriminate populations with a single modality is often limited. Typically, diseases are characterized by changes with small effect size in multiple domains; rarely, a single specific/sensitive feature fully characterizes individuals. While analyzing multiple MRI features potentially increases the power of phenotypic characterization, it aggravates statistical problems related to multiple comparisons. Methodologies designed to reduce the dimensions of information are imperative. A well‐known strategy is to aggregate voxels that represent a given structure in regions of interest (ROIs), resulting in a biologically comprehensive quantification. As manually drawing ROIs creates practical challenges (Tae, Kim, Lee, Nam, & Kim, 2008), automated methods for imaging segmentation of multiple contrasts represent a viable strategy (Faria, Liang, Miller, & Mori, 2017; Miller, Faria, Oishi, & Mori, 2013; Mori, Oishi, Faria, & Miller, 2013).

MRICloud (http://www.MRICloud.org) (Mori et al., 2016) is a recently developed web‐based tool with which to perform automated segmentation and quantification of multiple MRI modalities. MRICloud provides a platform to characterize anatomy (using T1 high‐resolution‐weighted images for volumetric analysis), white matter (using diffusion tensor images [DTI]), and resting‐state functional connectivity, built on structure‐based analysis. MRICloud can analyze all these modalities in the same anatomical framework, thus facilitating the integration of information from multiple domains in a biologically meaningful set of structures. In addition, MRICloud is a widely available tool, which is free online, completely automated and, therefore, meets the requirements for a neuroimaging tool that is widely applicable to large‐scale multimodal processing.

The reliability and accuracy of MRICloud for whole‐brain segmentation, based on DTI or T1‐WIs, have been extensively tested and validated (Ceritoglu et al., 2009; Liang et al., 2015; Oishi et al., 2008, 2009; Tang et al., 2015; Wu et al., 2016). A few other software that perform high‐resolution T1‐based automated segmentation, including FreeSurfer (Fischl, 2012), FSL (Jenkinson, Beckmann, Behrens, Woolrich, & Smith, 2012), SPM (Penny, Friston, Ashburner, Kiebel, & Nichols, 2007), ANTS (Avants et al., 2011), also underwent detailed reliability analysis, including testing the robustness of the respective pipelines to technical factors and artifacts (Ceritoglu et al., 2009; Han et al., 2006; Jovicich et al., 2009; Tustison et al., 2014; Ye et al., 2018). Most of these segmentation tools perform admirably when compared with the “gold standard” manual segmentation of selected structures, particularly when tested by the developers, in healthy subjects. A different aspect, less often reported, is the reproducibility of such technologies, over the entire brain, particularly when applied to DTI and resting‐state fMRI measurements, which has raised concerns about the interpretation of the results of these methods in the past (Huang et al., 2012; Morey et al., 2010; Shou et al., 2013; Vollmar et al., 2010).

Here, we assess the test–retest reproducibility of MRICloud structural quantification for different MRI modalities (T1‐based volumetric analysis, DTI for automated quantification of fractional anisotropy [FA] and mean diffusivity [MD], and resting‐state fMRI [rsfMRI] synchrony). We compare the MRICloud reproducibility with that of other well‐established methods, such as FreeSurfer and CONN‐SPM. Relevant information about biomarkers, particularly in longitudinal studies focused on subtle conditions, can be provided only by reliable neuroimaging tools and reproducible pipelines. It is the responsibility of the developers to provide users with the level of reproducibility of their tools, as the unknown reproducibility hinders the validation and the interpretation of the results.

## 3 MATERIALS AND METHODS

### 3.1 Participants and images

We used the two independent and public datasets to measure the reproducibility of MRICloud multimodality results:

- Kirby21, the “multimodal MRI reproducibility resource” (Landman et al., 2011). Kirby21 is a public dataset available in the Neuroimaging Informatics Tools and Resources Clearinghouse (http://www.nitrc.org). This database consists of 21 healthy volunteers with no history of neurologic diseases (11 male, 22–61 years old), scanned twice in a day, on a 3T Phillips Achieva Scanner. One subject (#8) was excluded because the original DTI scan was not available. A brief description of the image protocol follows: (a) T1‐weighted images: sagittal orientation, matrix 240 × 256 mm, voxel size 1 × 1 × 1.2 mm^3^, TR/TE/TI 6,300/3.1/842 ms, flip angle 8°; (b) diffusion tensor images (DTI): spin echo sequence, reconstructed matrix 256 × 256 mm, voxel size (interpolated to) 2.2 × 2.2 × 2.2 mm^3^, 65 slices, TE/TR 67/6,181 ms, flip angle 90°, 32 gradient directions, b‐factor = 700 s/mm^2^; (c) resting‐state functional MRI (rsfMRI): EPI sequence, voxel size 3 × 3 × 3 mm^3^, slice gap 1 mm, TR/TE 2,000/30 ms, flip angle 75°, voxel matrix 80 × 80 × 37, 210 frames per run.
- Human Connectome Project (HCP) test–retest dataset, which is a subset of the 1,200 individual MRIs, made public by HCP (Van Essen et al., 2013). It includes MRI test–retest MRIs of 45 healthy individuals (13 male, 22–35 years old), scanned in 3T machines, in variable intervals (4.7 ± 2 months interval, minimum = 1 month, maximum = 11 months). Two individuals have no rest–retest DTI. Note that the long retest interval increases likelihood of biological influences in the test–retest analysis, although these effects are presumably small in young healthy individuals. A brief description of the image protocol follows: (a) T1‐WI: axial orientation, FOV = 224 × 224 mm, voxel size 0.7 mm^3^ (isotropic), TR/TE/TI 2,400/2.14/1,000 ms, flip angle 8°; (b) DTI: 18 b0 images, 90 gradient directions, *b* = 3,000 s/mm^2^, TE/TR 89/5,520 ms, 1.25 mm voxel (isotropic); (c) rsfMRI: EPI sequence, voxel 2 mm^3^ (isotropic), TR/TE 720/33.1 ms, flip angle 52°, 72 slices, 1,200 frames per run.

### 3.2 Image processing

#### 3.2.1 Multimodality processing with MRICloud

The images were automatically postprocessed, segmented, and quantified in MRICloud (http://www.MRICloud.org) (Mori et al., 2016). Briefly, the process for segmenting the T1‐WI, used for volumetric analysis, involves orientation and homogeneity correction; two‐level brain segmentation (skull‐stripping, then whole brain); image mapping based on a sequence of linear, nonlinear algorithms, and large deformation diffeomorphic mapping (LDDMM); and a final step of multi‐atlas labeling fusion (MALF) (Tang et al., 2013), adjusted by PICSL (Wang & Yushkevich, 2013). Please read (Tang et al., 2015; Wu et al., 2016) for technical details. As for the multi‐atlas library, we chose “Adult_22_55yrs_283Labels_26atlases_M2_252_V9B” under MRICloud atlas choices. This atlaset contains 26 healthy individuals, 22–55 years old, demographically close to our cohort, as recommended for atlas mapping (Ye et al., 2018).

For the DTI, the tensor reconstruction and quality control followed the algorithm used by DtiStudio (http://www.MRIStudio.org). The automated DTI segmentation was similar to that used for T1‐WIs, except for the use of complementary contrasts (mean diffusivity [MD], fractional anisotropy [FA], and eigenvector [fiber orientation]) and a diffeomorphic likelihood fusion algorithm (Tang et al., 2014) for multi‐atlas mapping. Please read (Ceritoglu et al., 2009) for technical details. We used the only atlas library available for DTI mapping in MRICloud: “Adults_168labels_12atlases_V1,” which contains 12 healthy individuals, 20–50 years old.

For the rsfMRI postprocessing (Faria et al., 2012), the T1‐WI and the respective segmentations obtained as described above were coregistered to the motion and slice timing‐corrected, resting‐state dynamics. Time courses were extracted from all the cortical and subcortical gray matter regions defined in the atlases and detrended, regressed for motion and physiological nuisance (Behzadi, Restom, Liau, Liu, 2007). Intensity and motion “outliers” were extracted with ART (https://www.nitrc.org/projects/artifact_detect). Seed‐by‐seed correlation matrices were obtained from the “nuisance‐corrected” time courses, and z‐transformed by the Fisher's method.

After the multimodal brain segmentation and quantification, each individual, in each session, was represented by a vector of image features. The image features considered in this study were 226 structural volumes from T1‐WIs processing (listed in Table 1), 97 white matter structural FA, and 97 white matter structural MD measures from DTI processing (listed in Table 2), and 1,431 pairwise, resting‐state z‐correlations between 54 gray matter seeds from rsfMRI (listed in Table 3).

**Table 1:** Regional intraclass correlation coefficients (ICCs) for the T1‐based volumetric analysis, outputted from MRICloud (using Kirby21 and Human Connectome Project [HCP]) and FreeSurfer (using Kirby21)

	Mricloud	Kirby 21				Connectome				Kirby 21		
		L	R	White matter beneath		L	R	White matter beneath		Freesurfer	L	R
				L	R			L	R			
Cortex	Superior frontal gyrus	0.964	0.981	0.985	0.973	0.978	0.981	0.981	0.986	Superior frontal gyrus	0.947	0.954
	Superior frontal gyrus/pole	0.967	0.987	0.916	0.945	0.960	0.980	0.938	0.957			
	Superior frontal gyrus/prefrontal cortex	0.985	0.992	0.992	0.991	0.983	0.983	0.989	0.992			
	Middle frontal gyrus	0.986	0.987	0.970	0.979	0.988	0.979	0.994	0.987	Middle frontal gyrus	0.968	0.930
	Middle frontal gyrus/dorsolateral prefrontal cortex	0.989	0.993	0.984	0.988	0.985	0.992	0.982	0.984			
	Inferior frontal gyrus/pars opercularis	0.985	0.972	0.985	0.982	0.983	0.977	0.981	0.989	Opercular part of the inferior frontal gyrus	0.949	0.954
	Inferior frontal gyrus/pars orbitalis	0.986	0.994	0.977	0.980	0.979	0.989	0.967	0.978	Orbital part of the inferior frontal gyrus	0.612	0.646
	Inferior frontal gyrus/pars triangularis	0.989	0.978	0.983	0.987	0.979	0.982	0.984	0.989	Triangular part of the inferior frontal gyrus	0.961	0.878
	Rectus gyrus	0.973	0.975	0.955	0.964	0.990	0.990	0.954	0.977	Straight gyrus, gyrus rectus	0.654	0.833
	Middle fronto‐orbital gyrus	0.982	0.966	0.97	0.970	0.939	0.977	0.969	0.952			
	Lateral fronto‐orbital gyrus	0.978	0.989	0.986	0.951	0.987	0.984	0.981	0.980	Orbital gyri	0.922	0.958
	Postcentral gyrus	0.969	0.989	0.973	0.973	0.990	0.983	0.991	0.978	Postcentral gyrus	0.939	0.944
	Precentral gyrus	0.991	0.995	0.979	0.979	0.986	0.992	0.986	0.993	Precentral gyrus	0.950	0.942
	Superior parietal lobule	0.983	0.985	0.982	0.986	0.985	0.988	0.989	0.991	Superior parietal lobule	0.961	0.923
	Supramarginal gyrus	0.983	0.991	0.978	0.954	0.992	0.987	0.990	0.965	Supramarginal gyrus	0.958	0.787
	Angular gyrus	0.991	0.989	0.991	0.985	0.980	0.980	0.985	0.974	Angular gyrus	0.859	0.948
	Precuneus	0.985	0.985	0.98	0.979	0.985	0.984	0.975	0.979	Precuneus	0.914	0.924
	Cuneus	0.975	0.986	0.993	0.992	0.990	0.990	0.987	0.992	Cuneus	0.950	0.936
	Fusiform gyrus	0.989	0.987	0.990	0.987	0.995	0.990	0.989	0.990	Lateral occipito‐temporal gyrus (fusiform gyrus)	0.925	0.943
	Superior occipital gyrus	0.982	0.959	0.938	0.981	0.980	0.980	0.980	0.984	Superior occipital gyrus	0.939	0.912
	Middle occipital gyrus	0.993	0.991	0.987	0.995	0.996	0.992	0.992	0.992	Middle occipital gyrus	0.897	0.947
	Inferior occipital gyrus	0.992	0.987	0.971	0.972	0.988	0.982	0.972	0.981	Inferior occipital gyrus and sulcus	0.960	0.823
										Occipital pole	0.941	0.898
	Lingual gyrus	0.981	0.979	0.991	0.990	0.990	0.990	0.989	0.992	Lingual gyrus, ligual part of the medial occipito‐temporal gyrus	0.979	0.962
	Superior temporal gyrus	0.989	0.99	0.978	0.986	0.995	0.991	0.993	0.990	Lateral aspect of the superior temporal gyrus	0.965	0.959
										Planum temporale or temporal plane of the superior temporal gyrus	0.887	0.879
	Middle temporal gyrus	0.983	0.983	0.988	0.994	0.995	0.995	0.991	0.994	Middle temporal gyrus	0.939	0.961
										Anterior transverse temporal gyrus (of heschl)	0.823	0.871
	Middle temporal gyrus/pole	0.987	0.986	0.982	0.982	0.972	0.982	0.976	0.981	Temporal pole	0.760	0.659
	Superior temporal gyrus/pole	0.974	0.977	0.966	0.972	0.984	0.981	0.943	0.973			
	Inferior temporal gyrus	0.983	0.989	0.980	0.979	0.990	0.987	0.987	0.989	Inferior temporal gyrus	0.870	0.923
	Rostral anterior cingulate	0.977	0.986			0.984	0.970			Anterior part of the cingulate gyrus and sulcus	0.911	0.939
	Subcallosal anterior cingulate	0.936	0.919			0.937	0.945					
	Dorsal anterior cingulate	0.985	0.994	0.950	0.905	0.986	0.979	0.974	0.947	Middle‐anterior part of the cingulate gyrus and sulcus	0.863	0.915
	Subgenual anterior cingulate	0.953	0.918			0.964	0.965					
	Posterior cingulate	0.992	0.990	0.973	0.986	0.990	0.990	0.987	0.986	Middle‐posterior part of the cingulate gyrus and sulcus	0.940	0.887
										Posterior‐dorsal part of the cingulate gyrus	0.924	0.928
	Isthmus of the cingulate	0.966	0.960	0.966	0.960					Isthmus of the cingulate gyrus	0.715	0.935
	Insula	0.985	0.976	0.989	0.994					Short insular gyri	0.938	0.881
	Entorhinal area	0.919	0.869	0.916	0.838							
	Amygdala	0.960	0.961	0.974	0.973					Amygdala	0.846	0.902
	Parahippocampal gyrus	0.966	0.914	0.955	0.943					Parahippocampal gyrus, parahippocampal part of the medial occipito‐temporal gyrus	0.882	0.821
	Hippocampus	0.983	0.974	0.983	0.983					Hippocampus	0.927	0.960
	Body of the lateral ventricle	0.999	0.999	0.998	0.998					Lateral ventricle	0.997	0.999
	Frontal horn of the lateral ventricle	0.997	0.999	0.995	0.994							
	Lateral ventricle, atrium part	1.000	0.999	0.995	0.997							
	Occipital horn of the lateral ventricle	0.991	0.977	0.982	0.951							
	Inferior horn of the lateral ventricle	0.973	0.985	0.974	0.984					Inferior lateral ventricle	0.945	0.954
Ventricles and sulci	Sulci of the frontal lobe	0.99	0.976	0.990	0.991					Superior frontal sulcus	0.684	0.952
										Middle frontal sulcus	0.787	0.860
										Inferior frontal sulcus	0.945	0.812
										Orbital sulcus (h‐shaped sulci)	0.798	0.891
										Medial orbital sulcus (olfactory sulcus)	0.827	0.662
										Lateral orbital sulcus	0.701	0.551
										Fronto‐marginal gyrus (of wernicke) and sulcus	0.895	0.810
										Suborbital sulcus (sulcus rostrales, supraorbital sulcus)	0.658	0.670
										Transverse frontopolar gyri and sulci	0.808	0.831
	Central sulcus	0.983	0.989	0.974	0.955					Central sulcus	0.958	0.922
										Inferior part of the precentral sulcus	0.937	0.871
										Paracentral lobule and sulcus	0.900	0.911
										Postcentral sulcus	0.892	0.886
										Sulcus intermedius primus (of jensen)	0.702	0.839
										Superior part of the precentral sulcus	0.786	0.963
										Subcentral gyrus (central operculum) and sulci	0.829	0.953
	Sulci of the parietal lobe	0.989	0.980	0.989	0.988					Subparietal sulcus	0.893	0.947
										Intraparietal sulcus (interparietal sulcus) and transverse parietal sulci	0.852	0.87
										Parieto‐occipital sulcus (or fissure)	0.908	0.889
	Sulci of the occipital lobe	0.988	0.988	0.965	0.966					Middle occipital sulcus and lunatus sulcus	0.851	0.819
										Anterior occipital sulcus and preoccipital notch (temporo‐occipital incisure)	0.743	0.644
										Superior occipital sulcus and transverse occipital sulcus	0.956	0.779
										Calcarine sulcus	0.892	0.702
										Lateral occipito‐temporal sulcus	0.883	0.888
										Medial occipito‐temporal sulcus (collateral sulcus) and lingual sulcus	0.849	0.907
	Sulci of the temporal lobe	0.986	0.972	0.951	0.933					Inferior temporal sulcus	0.841	0.697
										Superior temporal sulcus (parallel sulcus)	0.963	0.862
										Transverse temporal sulcus	0.775	0.746
										Posterior transverse collateral sulcus	0.750	0.664
										Anterior transverse collateral sulcus	0.897	0.945
										Horizontal ramus of the anterior segment of the lateral sulcus (or fissure)	0.710	0.816
										Posterior ramus (or segment) of the lateral sulcus (or fissure)	0.925	0.861
										Vertical ramus of the anterior segment of the lateral sulcus (or fissure)	0.694	0.874
			
	Sulci of the cingulate gyrus	0.985	0.977	0.939	0.927					Marginal branch of the cingulate sulcus	0.693	0.844
										Pericallosal sulcus (of corpus callosum)	0.599	0.827
	Sylvian fissure and anterior insular sulcus	0.988	0.984	0.968	0.964					Anterior segment of the circular sulcus of the insula	0.814	0.854
										Inferior segment of the circular sulcus of the insula	0.944	0.878
										Superior segment of the circular sulcus of the insula	0.910	0.841
	Sylvian fissure and posterior insular sulcus	0.978	0.980	0.917	0.887					Long insular gyrus and central sulcus of the insula	0.735	0.558
Deep nucleae	Caudate nucleus	0.986	0.991	0.990	0.994					Caudate nucleus	0.988	0.966
	Globus pallidus	0.928	0.943	0.960	0.970					Pallidum	0.863	0.868
	Putamen	0.973	0.975	0.990	0.993					Putamen	0.781	0.969
	Thalamus	0.959	0.953	0.990	0.985					Thalamus	0.901	0.851
Deep white matter	Genu of corpus callosum	0.981	0.981	0.986	0.982					Anterior corpus callosum	0.87	
										Middle anterior corpus callosum	0.975	
	Body of corpus callosum	0.990	0.982	0.989	0.986					Central corpus callosum	0.974	
	Splenium of corpus callosum	0.972	0.990	0.996	0.997					Posterior corpus callosum	0.849	
										Middle posterior corpus callosum	0.969	
	Anterior limb of internal capsule	0.963	0.962	0.987	0.974							
	Posterior limb of internal capsule	0.930	0.937	0.971	0.981							
	Retrolenticular part of internal capsule	0.946	0.940	0.968	0.967							
	External capsule	0.934	0.955	0.980	0.980							
	Claustrum	0.852	0.468	0.800	0.800							
	Posterior thalamic radiation	0.936	0.965	0.996	0.992							
	Sagittal stratum	0.970	0.959	0.100	0.990							
	Superior fronto‐occipital fasciculus	0.766	0.766	0.816	0.776							
	Superior longitudinal fasciculus	0.94	0.971	0.990	0.990							
	Inferior fronto‐occipital fasciculus	0.937	0.948	0.960	0.967							
	Anterior corona radiata	0.971	0.972	0.993	0.994							
	Posterior corona radiata	0.910	0.939	0.978	0.985							
	Superior corona radiata	0.912	0.932	0.990	0.985							
	Corticospinal tract	0.927	0.932	0.919	0.918							
	Cerebral peduncle	0.935	0.940	0.970	0.980							
	Lateral part of the periventricular white matter	0.953	0.978	0.967	0.967							
	Fornix	0.912	0.894	0.900	0.880							
	Fornix/stria terminalis	0.939	0.787	0.942	0.953							
	Inferior cerebellar peduncle	0.858	0.922	0.950	0.920							
	Middle cerebellar peduncle	0.974	0.959	0.975	0.979							
	Superior cerebellar peduncle	0.917	0.868	0.942	0.920							
	Pontine crossing tract	0.735	0.803	0.869	0.888							
	Cerebellum gray matter	0.997	0.997	0.954	0.906					Cerebellum cortex	0.944	0.959
	Cerebellum white matter	0.978	0.969	0.983	0.983					Cerebellum white matter	0.942	0.929

**Table 2:** ICCs for regional fractional anisotropy (FA) and mean diffusivity (MD), outputted by MRICloud, using Kirby21 and HCP

MRICloud	Kirby 21				HCP			
	ICC for FA		ICC for MD		ICC for FA		ICC for MD	
	L	R	L	R	L	R	L	R
Superior parietal gyrus^a^	0.885	0.880	0.812	0.885	0.753	0.725	0.696	0.811
Cingulate gyrus^a^	0.808	0.709	0.878	0.938	0.804	0.796	0.800	0.824
Superior frontal gyrus^a^	0.925	0.850	0.901	0.856	0.796	0.669	0.489	0.474
Middle frontal gyrus^a^	0.842	0.809	0.883	0.788	0.798	0.635	0.432	0.625
Inferior frontal gyrus^a^	0.861	0.902	0.785	0.757	0.873	0.649	0.411	0.624
Precentral gyrus^a^	0.887	0.865	0.804	0.902	0.872	0.767	0.815	0.783
Postcentral gyrus^a^	0.959	0.928	0.918	0.894	0.712	0.735	0.774	0.816
angular gyrus^a^	0.774	0.863	0.726	0.883	0.732	0.710	0.691	0.773
Precuneus^a^	0.869	0.799	0.883	0.914	0.814	0.782	0.802	0.851
Cuneus^a^	0.907	0.764	0.832	0.777	0.851	0.756	0.795	0.822
Lingual gyrus^a^	0.625	0.691	0.655	0.753	0.762	0.715	0.615	0.744
Fusiform gyrus^a^	0.819	0.725	0.676	0.681	0.727	0.656	0.782	0.765
Superior occipital gyrus^a^	0.908	0.875	0.892	0.844	0.842	0.840	0.897	0.856
Inferior occipital gyrus^a^	0.825	0.839	0.506	0.740	0.825	0.707	0.855	0.769
Middle occipital gyrus^a^	0.888	0.841	0.785	0.795	0.821	0.723	0.869	0.829
Superior temporal gyrus^a^	0.416	0.604	0.623	0.727	0.851	0.714	0.775	0.776
Inferior temporal gyrus^a^	0.724	0.648	0.491	0.558	0.821	0.892	0.802	0.811
Middle temporal gyrus^a^	0.895	0.798	0.770	0.763	0.784	0.763	0.774	0.758
Lateral fronto‐orbital gyrus^a^	0.884	0.865	0.681	0.617	0.868	0.602	0.612	0.695
Middle fronto‐orbital gyrus^a^	0.786	0.787	0.512	0.406	0.713	0.785	0.687	0.635
Supramarginal gyrus^a^	0.896	0.937	0.875	0.885	0.876	0.770	0.766	0.794
rectus gyrus^a^	0.746	0.753	0.823	0.716	0.635	0.744	0.675	0.602
Insula^a^	0.847	0.887	0.964	0.671	0.804	0.796	0.777	0.734
Cerebellum	0.790	0.901	0.936	0.901	0.813	0.750	0.733	0.751
Corticospinal tract	0.897	0.858	0.741	0.659	0.886	0.897	0.886	0.893
Inferior cerebellar peduncle	0.436	0.649	0.478	0.588	0.800	0.853	0.836	0.819
Medial lemniscus	0.735	0.626	0.621	0.595	0.927	0.912	0.831	0.749
Superior cerebellar peduncle	0.642	0.781	0.605	0.607	0.797	0.756	0.791	0.775
Cerebral peduncle	0.817	0.814	0.785	0.404	0.921	0.935	0.714	0.880
Anterior limb internal capsule	0.755	0.659	0.653	0.616	0.891	0.803	0.552	0.797
Posterior limb internal capsule	0.818	0.846	0.427	0.716	0.868	0.895	0.641	0.814
Retro lenticular internal capsule	0.790	0.875	0.525	0.811	0.897	0.831	0.711	0.739
Posterior thalamic radiation	0.848	0.863	0.692	0.869	0.892	0.907	0.829	0.758
Anterior corona radiata	0.952	0.902	0.774	0.625	0.873	0.810	0.424	0.634
Superior corona radiata	0.910	0.941	0.715	0.861	0.924	0.875	0.683	0.729
Posterior corona radiata	0.928	0.886	0.647	0.838	0.867	0.950	0.828	0.894
Cingulum	0.915	0.907	0.750	0.730	0.878	0.810	0.679	0.859
Fornix stria terminalis	0.805	0.843	0.808	0.707	0.841	0.818	0.677	0.815
Superior longitudinal fasciculus	0.879	0.926	0.544	0.772	0.879	0.876	0.586	0.707
Superior fronto‐occipital fasciculus	0.760	0.851	0.822	0.872	0.760	0.677	0.822	0.453
Inferior fronto‐occipital fasciculus	0.828	0.912	0.641	0.453	0.891	0.848	0.710	0.720
Sagittal stratum	0.889	0.861	0.588	0.823	0.819	0.786	0.665	0.756
External capsule	0.802	0.860	0.455	0.710	0.854	0.758	0.477	0.684
Uncinated fasciculus	0.638	0.936	0.696	0.767	0.807	0.881	0.785	0.811
Pontine crossing tract	0.723	0.882	0.693	0.602	0.881	0.905	0.858	0.866
Middle cerebellar peduncle	0.677	0.775	0.312	0.284	0.829	0.757	0.845	0.864
Fornix	0.893	0.718	0.946	0.703	0.774	0.875	0.602	0.676
Genu corpus callosum	0.896	0.923	0.574	0.617	0.901	0.850	0.554	0.641
Body of the corpus callosum	0.878	0.914	0.922	0.830	0.898	0.900	0.598	0.828
Splenium corpus callosum	0.880	0.919	0.736	0.894	0.866	0.920	0.686	0.770

Abbreviations: HCP, Human Connectome Project; ICC, intraclass correlation coefficients.

a White matter labels beneath the gray matter.

**Table 3:** Averaged ICCs of seed‐by‐seed correlations outputted by the rsfMRI processed with MRICloud (using Kirby21 and HCP) and SPM CONN (using Kirby21)

Structure	ICC for Mricloud				ICC for SPM CONN	
	Kirby 21		HCP		Kirby 21	
	L	R	L	R	L	R
Angular gyrus	0.387	0.376	0.482	0.527	0.155	0.264
Cuneus	0.433	0.438	0.392	0.417	0.153	0.080
Fusiform gyrus	0.342	0.419	0.253	0.339	0.163	0.249
Rectus gyrus	0.384	0.340	0.444	0.458	0.150	0.077
Inferior frontal gyrus/pars opercularis	0.243	0.369	0.470	0.432	0.228	0.238
Inferior frontal gyrus/pars orbitalis	0.283	0.413	0.417	0.449	0.092	0.208
Inferior frontal gyrus/pars triangularis	0.302	0.367	0.350	0.442	0.183	0.103
Inferior occipital gyrus	0.356	0.355	0.381	0.396	0.156	0.282
Inferior temporal gyrus	0.329	0.387	0.362	0.446	0.130	0.107
Lingual gyrus	0.374	0.394	0.334	0.304	0.219	0.150
Middle frontal gyrus	0.317	0.418	0.435	0.532	0.270	0.216
Middle frontal gyrus (dorsolateral prefrontal cortex)	0.258	0.378	0.421	0.460	0.124	0.216
Middle occipital gyrus	0.332	0.447	0.342	0.338	0.189	0.246
Middle temporal gyrus	0.323	0.400	0.443	0.446	0.276	0.129
Middle temporal gyrus/pole	0.402	0.337	0.465	0.397	0.116	0.025
Postcentral gyrus	0.388	0.380	0.327	0.366	0.176	0.166
Posterior cingulate cortex	0.363	0.364	0.385	0.189	0.147	0.213
Precentral gyrus	0.424	0.378	0.333	0.393	0.223	0.164
Precuneus	0.425	0.443	0.412	0.386	0.135	0.270
Superior frontal gyrus	0.372	0.406	0.415	0.444	0.133	0.185
Superior frontal gyrus/pole	0.413	0.416	0.280	0.388	0.012	0.000
Superior frontal gyrus/prefrontal cortex	0.356	0.328	0.431	0.469	0.195	0.202
Superior occipital gyrus	0.425	0.434	0.030	0.188	0.253	0.204
Superior parietal lobule	0.284	0.261	0.387	0.366	0.229	0.128
Superior temporal gyrus	0.364	0.350	0.333	0.383	0.240	0.178
Superior temporal gyrus/pole	0.413	0.391	0.456	0.399	0.171	0.185
Supramarginal gyrus	0.316	0.382	0.445	0.437	0.233	0.178

Abbreviations: HCP, Human Connectome Project; ICC, intraclass correlation coefficients.

#### 3.2.2 T1‐WIs volumetric analysis with FreeSurfer

Volumes of Kirby21 cortical labels and the deep gray matter were obtained from FreeSurfer v.5.3, for further comparison of reliability with MRICloud. Briefly, images are aligned to the Talairach and Tournoux atlas, corrected for magnetic field inhomogeneity, skull‐stripped, and the tissues are classified as gray matter, white matter, or CSF. Next, the white surface (the interface between gray and white matter) and the pial surfaces are estimated by triangle meshes and smoothed with a Gaussian filter of 10 mm FWHM (Fischl & Dale, 2000). Cortical thickness is calculated as the shortest distance between the pial and white surface at each vertex across the cortical mantle. The cortical volume is the multiplication of cortical thickness and surface area. The volumes for subcortical regions are calculated as well (Fischl et al., 2002). For comparison of reliability, we determined the correspondence between MRICloud and FreeSurfer labels (listed in Table 1), which is feasible since both methods label according to structural anatomy.

#### 3.2.3 rsfMRI analysis with SPM CONN toolbox

We used the SPM CONN toolbox, version 17e, to preprocess and perform first‐level statistics of Kirby21 rsfMRI data, and further compared the reliability of these results with those from MRICloud. The CONN toolbox is the most widely used tool for processing rsfMRI and uses a combination of SPM12 and native‐implemented functions. The preprocessing was attuned to keep the rsfMRI in the native space and used the default CONN parameters for slice‐time correction and realignment. As in the MRICloud pipeline, ART identified the outlier scans (97th percentiles in a normative sample). The effect of the rest model and its first‐order derivative were used as first‐level covariates (individual regressors). Sequentially, the processed functional images were detrended and band‐pass‐filtered (0.008–0.09 Hz). After tissue segmentation and skull‐stripping, the T1‐WIs and the respective parcellation maps obtained from MRICloud were brought to the rsfMRI space. The use of the same anatomic labels enabled a direct comparison between SPM CONN and MRICloud seed‐by‐seed correlations.

Although there are descriptions of automated white matter parcellations using cortical‐parcellation‐based strategies and fiber clustering parcellation (Zhang et al., 2019), to the best of our knowledge, MRICloud is the only automated pipeline available for whole‐brain, DTI structure‐based analysis. Therefore, the reproducibility of DTI regional quantification outputted from MRICloud was not directly compared with that from other software.

### 3.3 Statistical analysis

#### 3.3.1 Test–retest reproducibility

To assess the test–retest reliability of the different metrics in each region of interest, we used the intraclass correlation coefficient (ICC) (Shrout & Fleiss, 1979). To access a global measure of the reproducibility for a given modality, we used the image intraclass correlation coefficient (I2C2) (Shou et al., 2013), which takes in account the total variability of the data. Because I2C2 is less sensitive to regions with low variability, it tends to be lower, and more realistic, than the average of regional ICCs.

A problem that cannot be intuitively solved by looking at I2C2 or ICCs is whether the global individual pattern of image features is reproducible. This “fingerprint” problem has recently been explored in neuroimaging (Finn et al., 2015; Liu, Liao, Xia, & He, 2018; Mars et al., 2018). Assuming that the MRI data itself are reproducible (which is a valid assumption for structural data, such as T1‐based volumes), if the postprocessing and quantification tool is reliable, a given individual will be closer to him/herself rather than to someone else in the space of the image features.

In order to explore this idea, we used principal component analysis (PCA) to reduce the dimensionality of the data in each modality. The first three principal components, which are linear combinations of the features in question, are those that explain most of the data variability. The distances across different subjects in the 3D PCA plots (indicated by circles with different colors in our figures) reflect anatomical variability among normal brains, as well as the measurement variability. Through the test–retest pairs (indicated by circles of same colors in our figures), one can estimate the size of the measurement variability, that is, the precision of the measurement, with respect to the anatomical variability of the population. We also ranked the Euclidean distance among subjects in the three‐dimensional PCA space. The lowest rank of 1 represents a pair of individuals that are closest in the feature space (i.e., a pair with the lowest variability). If the measurement variability is lower than the anatomical variability, a test–retest pair has a low score, ideally, 1. We judged “correct classification” when the two closest neighbors were the first and second scans of the same subject (the “test–retest” pair), and “misclassification” otherwise.

Finally, we checked for significant differences in the diverse metrics between groups (test and retest) using Wilcoxon, corrected for multiple comparisons with false discovery rate. We also calculated the percentage of difference between the test and retest metrics, as ([test metric + retest metric]/test metric) * 100. Furthermore, although this study is not designed to test the reliability of the segmentation itself, we calculated the Dice index between each pair (test–retest) of parcels obtained by the T1 and DTI processing in MRICloud, as a sanity check. Note that the Dice was the only metric calculated not in the native space, but in a MNI space, to ameliorate differences in the head position between the test–retest scans.

#### 3.3.2 Power analysis: illustrating the effect of the data variability

Power analysis was used to illustrate the effects of data variability (both biological and technical) on the automated imaging quantification. For a proof of concept, we chose two regions (one with a large ICC and the other with a low ICC), from the volumetric T1‐based analysis and from the DTI analysis. We calculated the sample size necessary to detect group differences at an alpha of 0.05 and a power of 0.8, using GPOWER (http://www.gpower.hhu.de/). The sample size that resulted was inversely proportional to the data variability, which was inversely proportional to the ICC.

## 4 RESULTS

### 4.1 Volumetric (T1‐based) test–retest reliability

**Figure 1:**
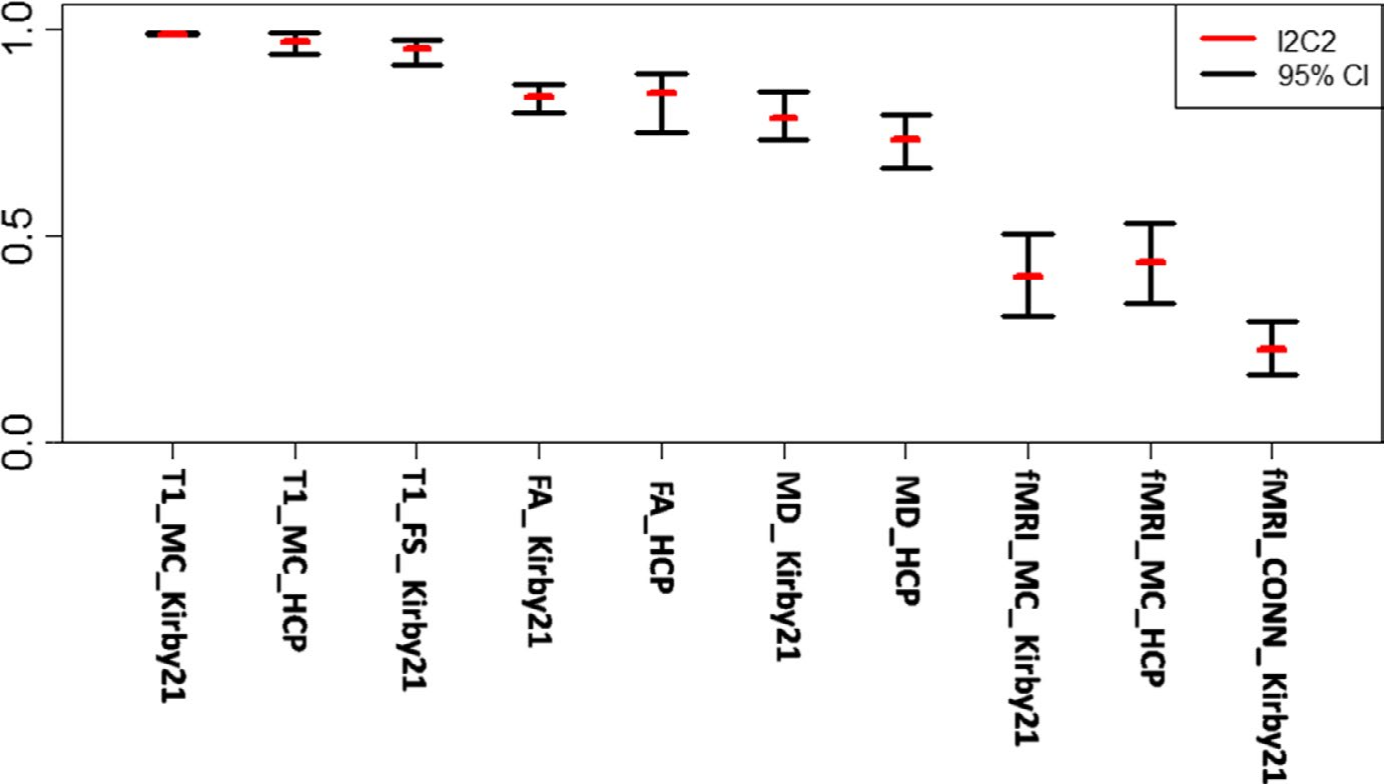
I2C2 for the results of T1‐volumetric analysis, fractional anisotropy (FA), and mean diffusivity (MD) from DTI, and resting‐state fMRI, in two independent datasets (Kirby21 and HCP), using different platforms (MRICloud [MC], FreeSurfer [FS], Connectivity toolbox in SPM [CONN‐SPM])

**Figure 2:**
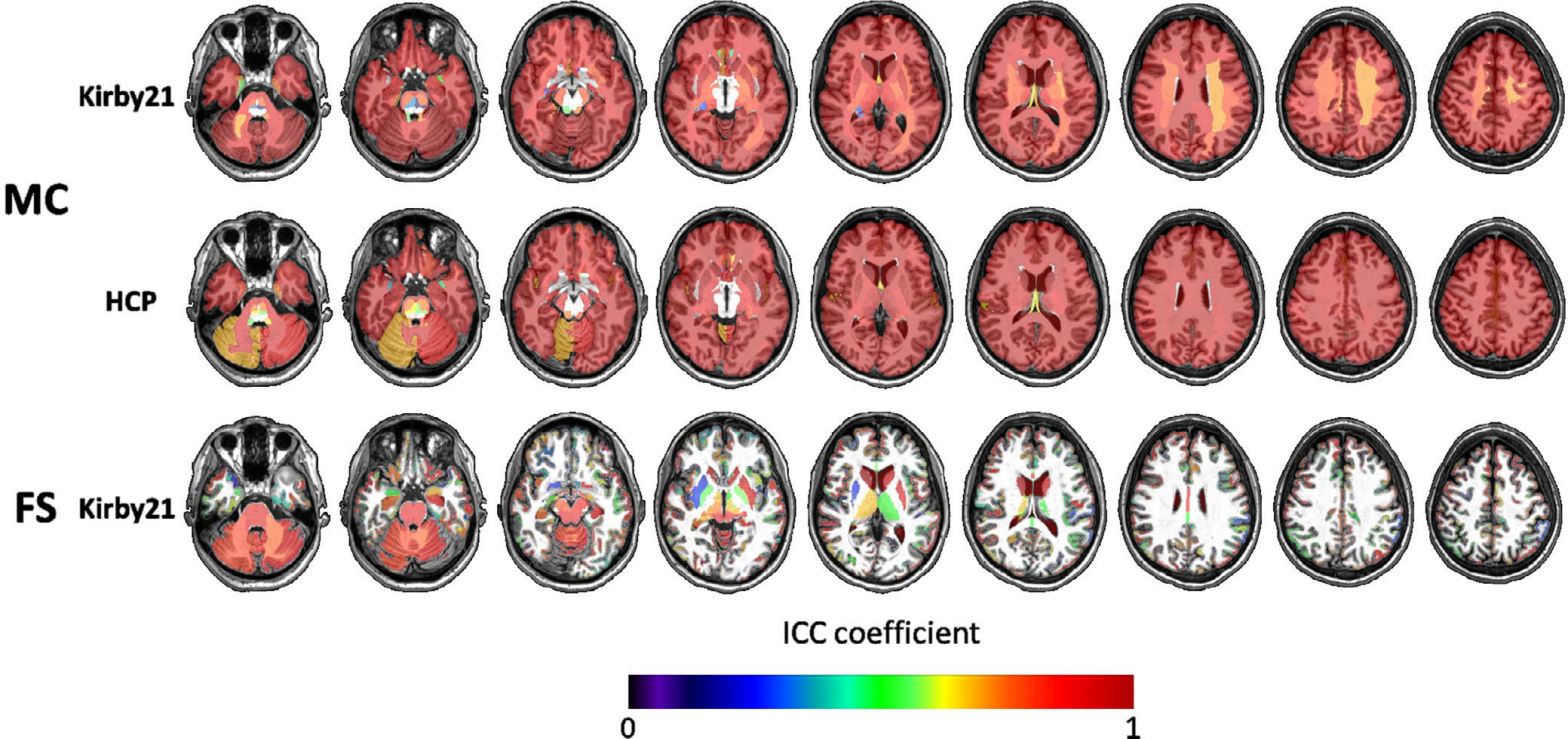
Color‐coded regional ICCs for the volumetric outputs of MRICloud (MC) and FreeSurfer (FS), in two independent datasets (Kirby21 and HCP), overlaid on a representative brain

The global I2C2 coefficients for the MRICloud volumetric analysis were very high (1 indicating perfect agreement): HCP dataset: 0.989 (confidence interval, CI: 0.987–0.992) and 0.936 (CI: 0.870–0.998), Kirby21 dataset: 0.988 (CI: 0.982–0.991) and 0.997 (CI: 0.995–0.999), for the cerebral cortex and deep gray matter, respectively (Figure 1). The ROIs showed consistently high ICCs (Table 1, Figure 2), including those in the white matter, which we were able to obtain because MRICloud performs whole‐brain parcellation. Only a few small parcels, primarily in the brainstem, had ICCs below 0.9, and no region had an ICC below 0.8.

Compared to MRICloud, the I2C2s for Kirby21 were slightly lower for the FreeSurfer results: 0.920 (CI: 0.871–0.951) for the cerebral cortex and 0.967 (CI: 0.933–0.988) for the deep gray matter. The regional ICCs were also lower, in general (Figure 2, bottom), with a few regions, particularly at the deep gray matter, showing an ICC of approximately 0.8.

**Figure 3:**
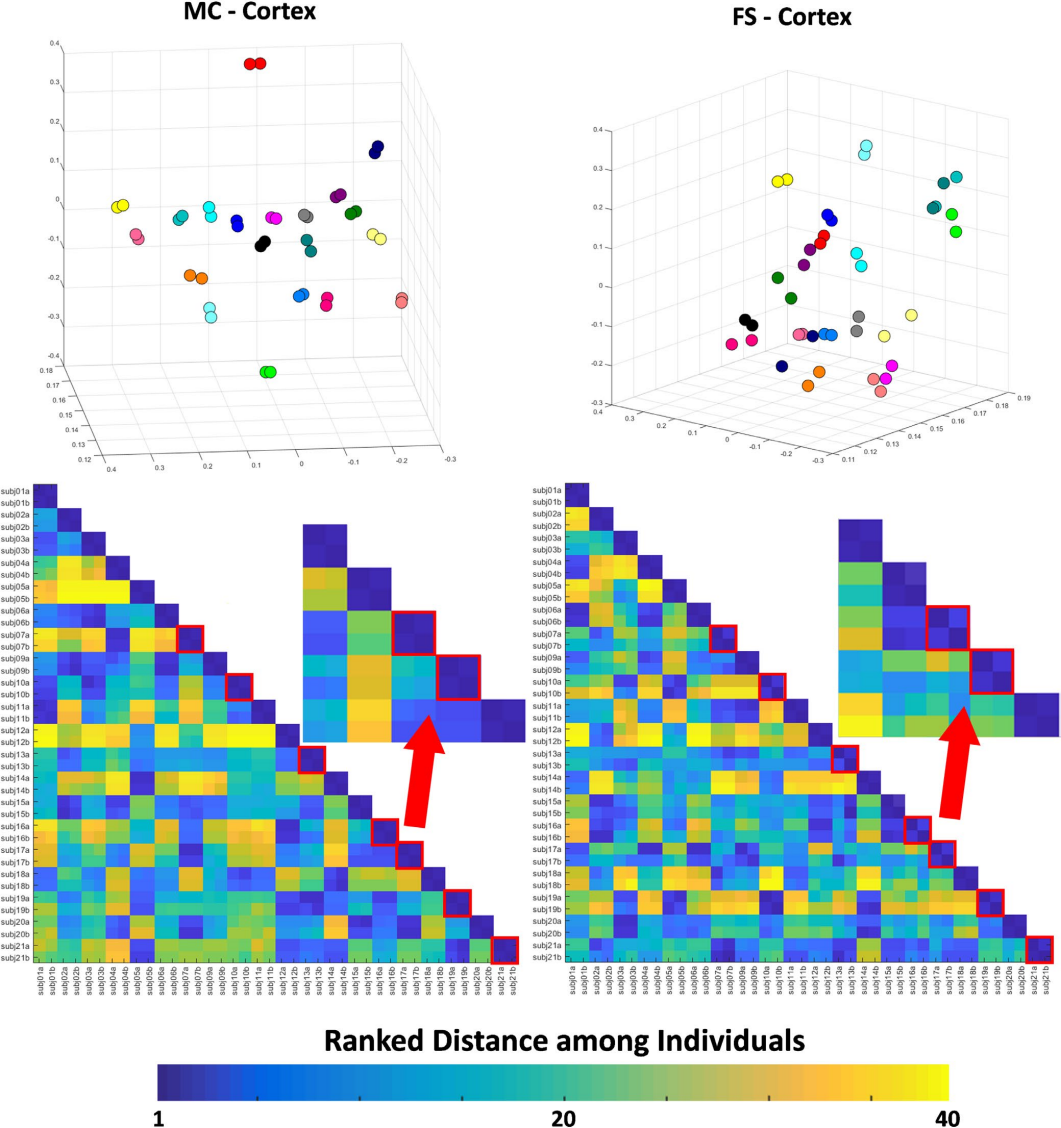
Top: 3D PCA plot created with the volumes of Kirby21 cortical areas, outputted by MRICloud (MC) and FreeSurfer (FS). Individuals are color‐coded, that is, the same color represents a “test–retest” pair. Bottom: matrix of ranked distance between individuals in the three‐dimensional PCA plot. If the variance in the measurement between scan sections was minimal, a test–retest pair was scored 1 (dark blue). Test–retest pairs that scored higher than 1 (i.e., the individual was closer to someone else rather than him/herself in the second scan) are framed in red

**Figure 4:**
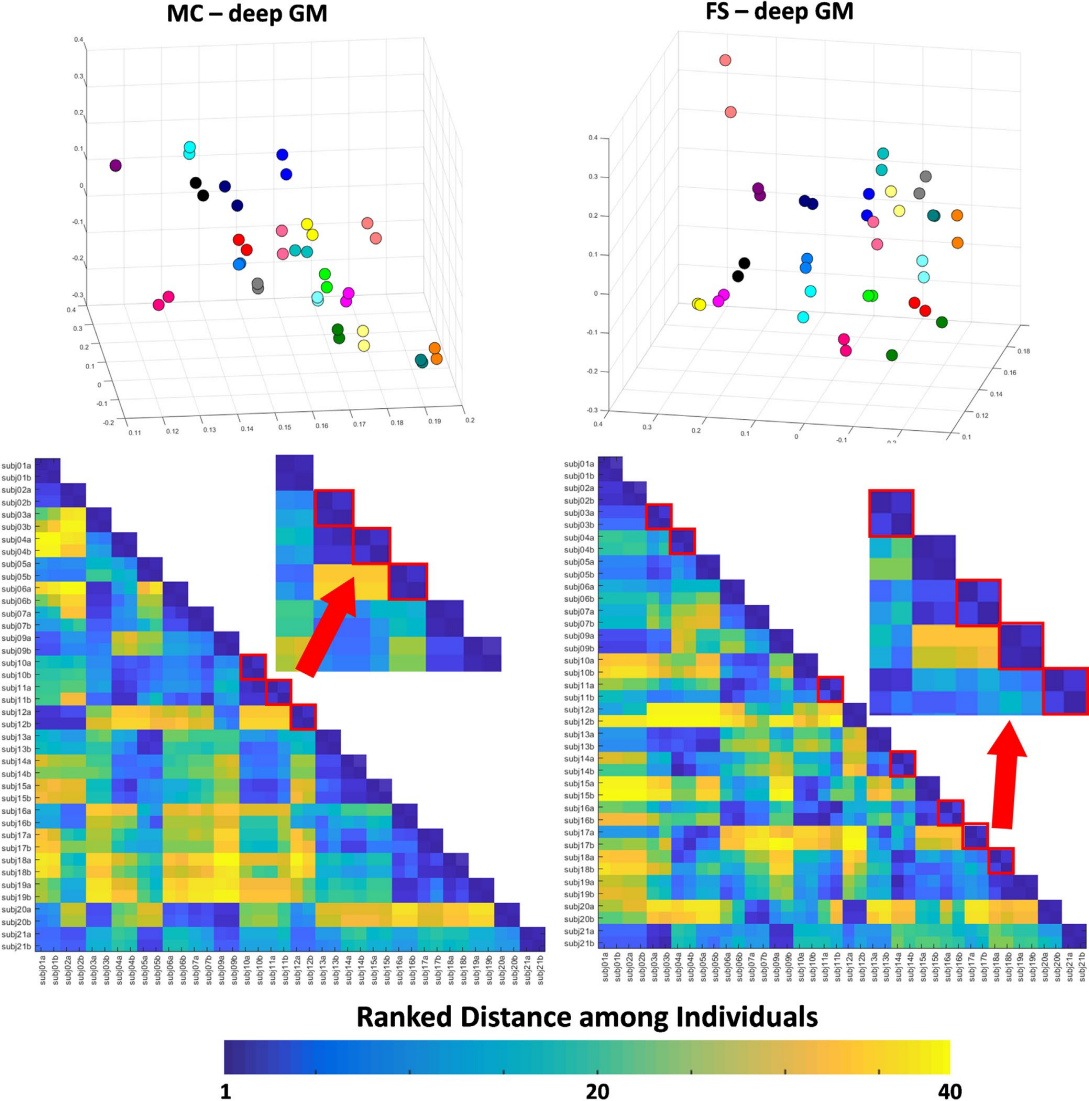
Top: 3D PCA plot created with the volumes of the Kirby21 deep gray matter areas, outputted by MRICLoud (MC) and FreeSurfer (FS). Individuals are color‐coded; that is, the same color represents a “test–retest” pair. Bottom: matrix of ranked distance between individuals in the three‐dimensional PCA plot. If the variance in the measurement between scan sections was minimal, a test–retest pair was scored 1 (dark blue). Test–retest pairs that scored higher than 1 (i.e., the individual was closer to someone else rather than to him/herself in the second scan) are framed in red

The three‐dimensional PCA plot (Figure 3, top) for Kirby21 data shows that the measurement variability was higher for the results of FreeSurfer (average Euclidian distance between the test–retest pair 0.043 ± 0.025) compared to those from MRICloud (average Euclidian distance between the test–retest pair 0.016 ± 0.007 for Kirby21 and 0.016 ± 0.010 for HCP), while still lower than that of the anatomical variability in both cases. Similar results were obtained using the deep gray matter volumes (Figure 4, top). Again, the measurement variability for deep gray matter volumes was higher for the results of FreeSurfer (average Euclidian distance between the test–retest pair 0.042 ± 0.033) compared to those from MRICloud (average Euclidian distance between the test–retest pair 0.028 ± 0.017 and 0.028 ± 0.073 for Kirby21 and HCP respectively), while still lower than that of the anatomical variability in both cases.

This idea was reinforced by the ranked distance matrix (Figures 3 and 4, bottom). For the results of MRICloud, individuals in the test–retest pair were always the closest (ranked distance of 1) when using cortical volumes (Figure 3, bottom right), or almost always the closest (except by one case), when using the deep gray matter volumes (Figure 4, bottom right). There were 7 “misclassifications” (i.e., the closest individual in the first scan was not him/herself in the second scan) when using the volumetric results of FreeSurfer, both for the superficial and for the deep gray matter (Figures 3 and 4, bottom left).

There were no significant differences in regional volumes, as outputted by MRICloud, between the test and the retest sets. The average difference between the test and retest volumes was 1.76% for Kirby21, and 2.8% for HCP. The Dice indices between pairs (test–retest) of parcels were high 0.814 ± 0.141 for Kirby21 and 0.8 ± 0.097 for HCP.

### 4.2 DTI test–retest reliability

**Figure 5:**
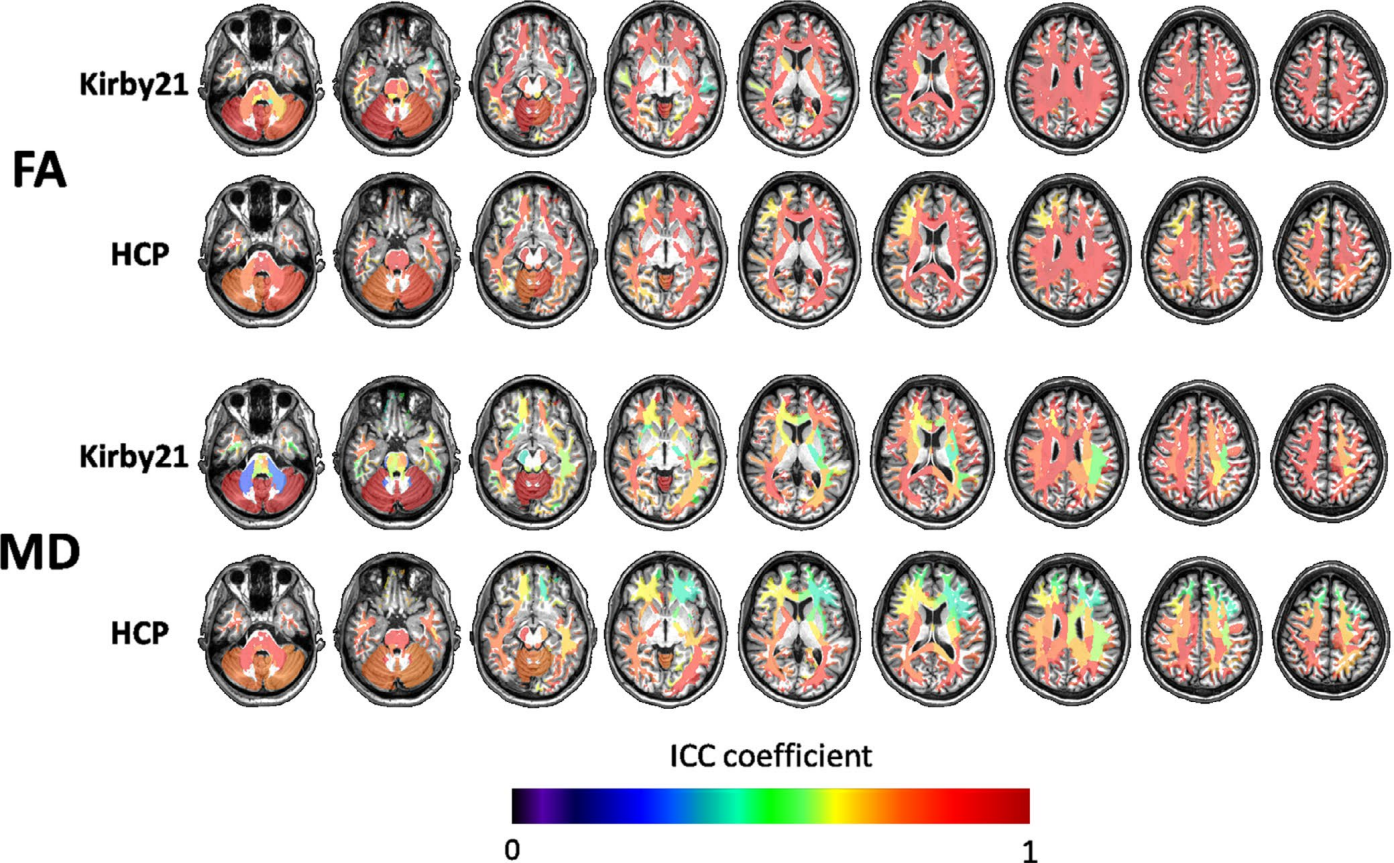
Color‐coded regional ICCs for the DTI outputs of MRICloud (FA, fractional anisotropy, MD, mean diffusivity) in two independent datasets (Kirby21 and HCP), overlaid on a representative brain

The global I2C2 coefficients for the MRICloud analysis of the fractional anisotropy (FA) and mean diffusivity (MD) were as follows: Kirby21: 0.836 (CI: 0.798–0.869) and 0.787 (CI: 0.735–0.851), respectively; HCP: 0.844 (CI: 0.751–0.892) and 0.733 (CI: 0.666–0.793), respectively (Figure 1). The regional ICCs (Table 2, Figure 5) were higher for FA than for MD. The FA ICCs were virtually higher than 0.8, while, for MD, a few regions scored below this level, particularly in the brainstem. In contrast to the volumetric analysis, there was more variation on the ICCs, with some areas scoring high (ICC > 0.9) and others low (ICC < 0.5).The cerebellar peduncles had the lowest ICCs.

**Figure 6:**
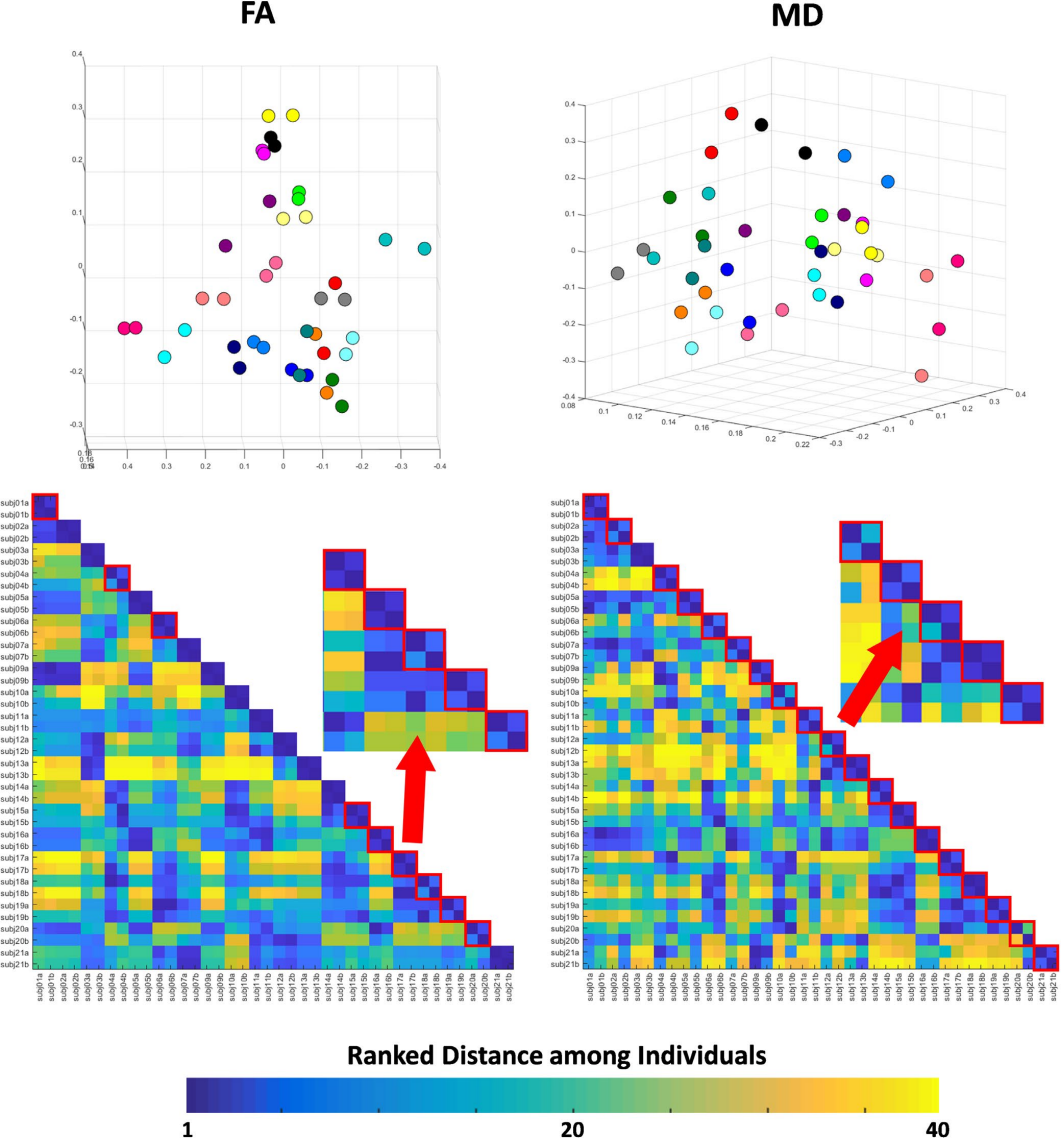
Top: 3D PCA plot created with the Kirby21 regional measures of fractional anisotropy (FA) and mean diffusivity (MD). Individuals were color‐coded; that is, the same color represents a “test–retest” pair. Bottom: matrix of ranked distance between individuals in the three‐dimensional PCA plot. If the variance in the measurement between scan sections was minimal, a test–retest pair scored 1 (dark blue). Test–retest pairs that scored higher than 1 (i.e., the individual was closer to someone else rather than to him/herself in the second scan) are framed in red

Although higher than in the volumetric analysis, the measurement variability for Kirby21 FA and MD (the distance between the rest–retest individuals, or dots with the same color in the PCA plots of Figure 6) was, on average, lower than the anatomical variability (the distance among different individuals). The measurement variability was higher for MD (average Euclidian distance between the test–retest pair 0.147 ± 0.079 and 0.119 ± 0.072 for Kirby21 and HCP, respectively) than for FA (average Euclidian distance between the test–retest pair 0.060 ± 0.039 and 0.057 ± 0.032 for Kirby21 and HCP, respectively). Again, the ranked distance matrices offered a different view of the same findings. Using the FA metrics, individuals in a test–retest pair were the closest (ranked distance of 1) in the majority of cases (Figure 6, bottom right), although there were 9 “misclassifications” (i.e., the closest individual in the first scan was not him/herself in the second scan). Using MD, individuals in the test–retest pair were often not the closest (Figure 6, bottom left, 19 “misclassifications”).

There was no significant difference in FA or MD between the test and the retest sets. The difference between the test and retest metrics was 0.64% for FA and 1.79% for MD, in Kirby21, and 0.5% for FA and 1.9% for MD, in HCP. The Dice indices between pairs (test–retest) of parcels were high (0.896 ± 0.05 and 0.838 ± 0.066 for Kirby21 and HCP, respectively).

### 4.3 rsfMRI test–retest reliability

**Figure 7:**
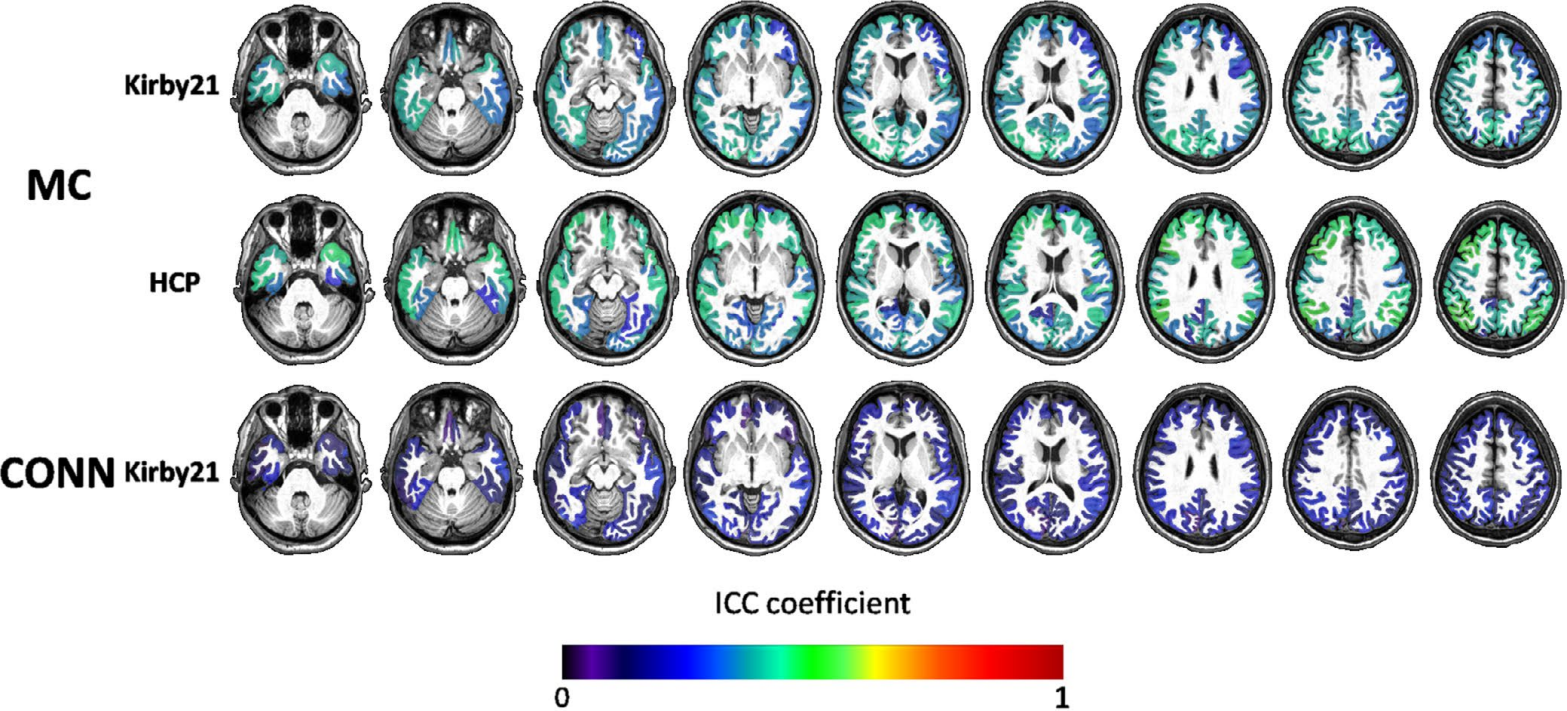
Color‐coded regional mean ICCs for the resting‐state fMRI outputs of MRICloud (MC) and CONN‐SPM, in two independent datasets (Kirby21 and HCP), overlaid on a representative brain, overlaid on a representative brain

The rsfMRI showed the lowest global I2C2 and regional ICCs among all the tested modalities. The global I2C2 for the MRICloud outputs (z‐transformed correlation among pairs of cortical seeds) was 0.437 (CI: 0.337–0.530) in HCP and 0.403 (CI: 0.309–0.507) in Kirby21. For the SPM CONN outputs, the I2C2 was 0.227 (CI: 0.164–0.293) in Kirby21 (Figure 1). The ICC for a given label was calculated as the mean of the ICCs for correlations between that given seed to each other seed (Table 3). The maximum ICC for a parcel using the MRICloud outputs did not exceed 0.6, with the majority of ICCs fluctuating around 0.4 (Figure 7). For the SPM CONN processing, the maximum ICC did not exceed 0.5, with the majority of ICCs fluctuating around 0.25.

**Figure 8:**
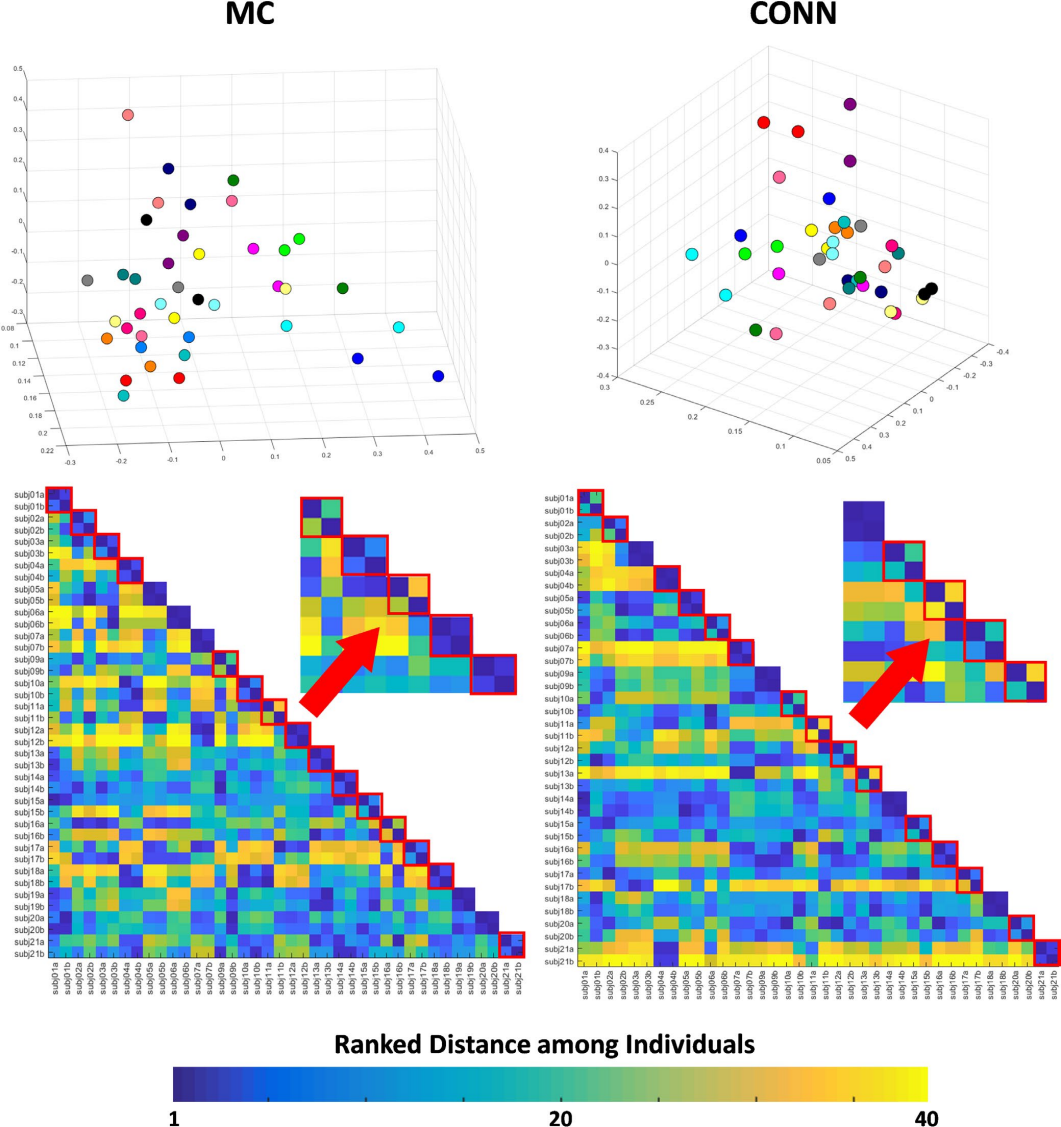
Top: 3D PCA plot created with z‐transformed correlations between the Kirby21 fMRI time courses of a pair of seeds, outputted by MRICloud (MC) and SPM CONN. Individuals were color‐coded, that is, the same color represents a “test–retest” pair. Bottom: matrix of ranked distance between individuals in the three‐dimensional PCA plot. If the variance in the measurement between scan sections was minimal, a test–retest pair scored 1 (dark blue). Test–retest pairs that scored higher than 1 (i.e., the individual was closer to someone else rather than to him/herself in the second scan) are framed in red

The measurement variability, or the distance among test–retest pairs (same color dots) in the PCA plots created with the pairwise z‐rsfMRI correlations (Figure 8, top), was lower, on average, than the anatomical/functional variability, or the distance among different individuals, although the variability was seemingly higher than that obtained for volumes or DTI metrics.The ranked distance among Kirby21 individuals (Figure 8, bottom) showed predominantly “misclassifications” (the closest individual in the first scan was not him/herself in the second scan): 15 for MRICloud, 15 for CONN‐SPM. The individual variability was still lower for MRICloud (average Euclidian distance between the test–retest pair 0.163 ± 0.093 and 0.134 ± 0.090 for Kirby21 and HCP, respectively) compared to CONN‐SPM (average distance between the test–retest pair 0.175 ± 0.131).

There were no significant differences in the outputs of MRICloud between the test and the retest sets.

### 4.4 Power

The power analysis illustrated the effects of data variability on the automated imaging quantification. For proof of concept, we chose regions with the highest and the lowest test–retest reliability, as measured by ICCs. The power analysis (Table 4) showed that the volumetric data were very stable, meaning that there is a small effect size among scan sets and that thousands of subjects would be needed to detect differences between them. The results are even more drastic for regions with a high ICC (e.g., precentral gyrus > globus pallidus). For the DTI analysis, here represented by the fractional anisotropy, the results were the same for highly reproducible areas (e.g., projection fibers at the pons level), namely, a small effect size between examinations, and a large sample needed to detect differences between them. However, as the range of ICCs was wider compared to volumes in areas with low ICCs (e.g., inferior cerebellar peduncle), the effect size between scans was relatively high (0.69) and less than 100 patients would be needed to detect differences between the scan sets. As this uses a test–retest design, these differences are technical, rather than biological.

**Table 4:** Power analysis illustrated for regions with high and low ICCs in the T1‐volumetric analysis and DTI quantification performed with MRICloud

	T1‐based volumes	Fractional anisotropy (FA) – DTI
High ICC area	Precentral (ICC = 0.99) *d* = 0.04/*n* = 20,260	Projection fibers at pons level (ICC = 0.89) *d* = 0.1/*n* = 3,234
Low ICC area	Globus pallidus (ICC = 0.92) *d* = 0.06/*n* = 8,072	Inferior cerebellar peduncle (ICC = 0.43) *d* = 0.69/*n* = 70

Abbreviations: DTI, diffusion tensor images; ICC, intraclass correlation coefficients.

## 5 DISCUSSION

We test–retested an automated web‐based tool (MRICloud) that performs segmentation and quantification of multimodality MRI (volume from T1‐WIs and FA, and MD from DTI and rsfMRI seed‐by‐seed synchrony). The reproducibility rivaled, or was slightly superior, to that from other well‐established methods (FreeSurfer, SPM CONN). As discussed in detail below, the reproducibility was (a) globally very high for T1‐volumetric analysis; (b) high for DTI analysis, but regionally more variable than for T1‐volumetric analysis; and (c) globally low for rsfMRI. To shed light on the reproducibility of postprocessing and quantification tools for MRI is essential, particularly when, by their nature (automated, user‐friendly), these tools are used for processing data on a large scale.

### 5.1 Reproducibility of volumetric quantification

The I2C2 and the regional ICCs were high for the volumetric analysis (vast majority > 0.9, while perfect agreement is 1), reflecting the high stability of the volumetric data and suggesting that subtle differences appointed by them are reliable (Wonderlick et al., 2009). Although we observed a tendency of small areas to have lower ICCs than large areas, the small variation of ICCs prevented the determination of a significant relationship between the ROI volume and the respective reproducibility of its volume measures. We found reproducibility similar to that in previous studies for data processed with FreeSurfer (Morey et al., 2010; Wonderlick et al., 2009) using different scanners, inclusion criteria, scan–rescan intervals, and software versions, indicating that the stability of T1‐based volumetric analysis overcomes all these factors.

**Figure 9:**
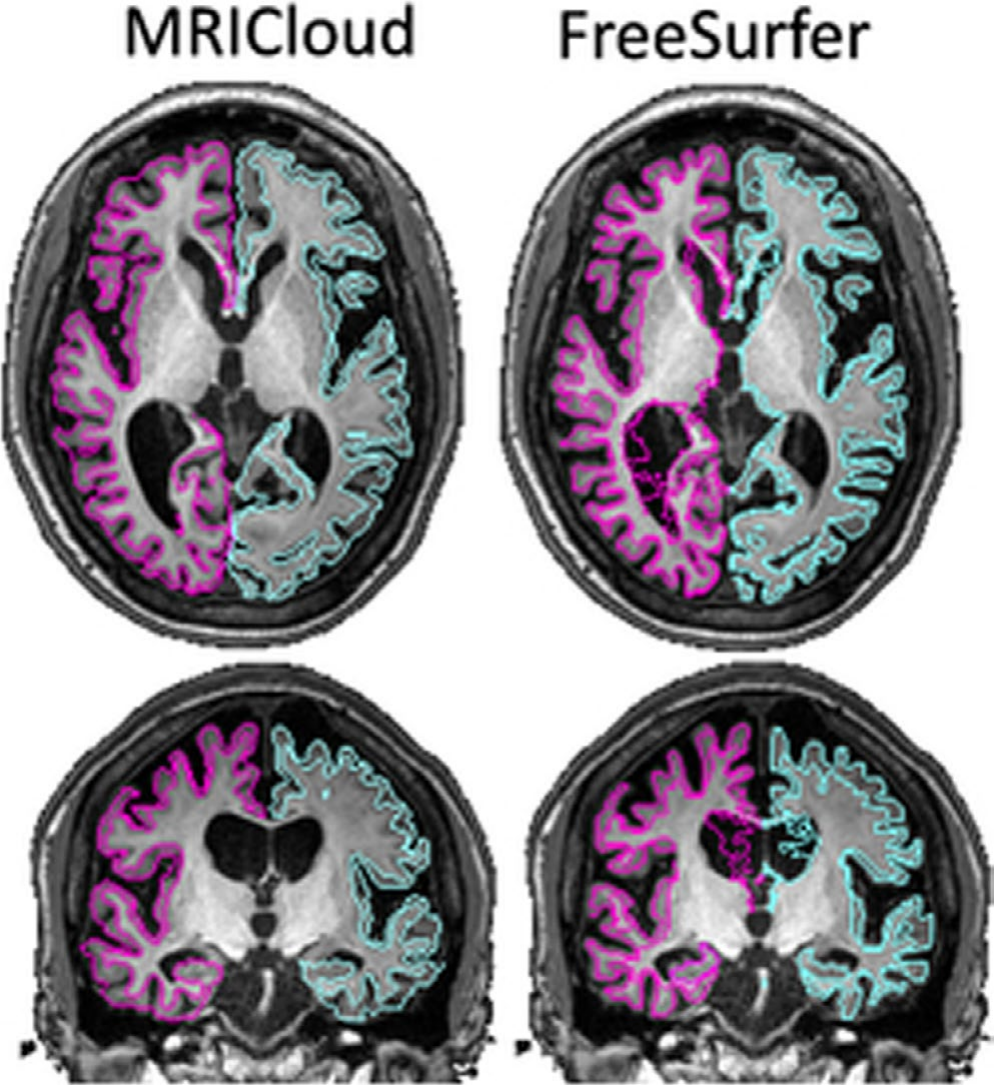
Segmentation of cortex and white matter outputted from MRICloud (left) and FreeSurfer (right) of a brain with large degree of atrophy

Both MRICloud and FreeSurfer had extremely high ICCs for volumetric analysis (0.98 vs. 0.92), which indicates very high reproducibility, suggesting both methods perform adequately in anatomically normal data. Nevertheless, understanding the source of ICCs variability can lead to improvements in data postprocessing by identifying factors (such as the parcellation scheme, mapping algorithm or set of atlases) that may impact reproducibility. For instance, MRICloud uses diffeomorphic mapping (LDDMM) and multiple atlases, with a large range of anatomical variability. This makes the method effective on both normal brains and brains with a large range of nonlocalized deformations (“atrophy‐like”), while methods that assume a stable, healthy pattern may perform worse on these scenarios {Oishi, 2009 #1831}. This is illustrated in Figure 9, where MRICloud outputted a qualitatively reasonable segmentation for an individual with marked brain atrophy, due to hereditary spastic paraplegia type 11.

### 5.2 Reproducibility of DTI results

The I2C2s for DTI‐derived data, processed with MRICloud, were high, while lower than those from the volumetric analysis. Although most of the previous studies looked at DTI reliability on different scans and/or from multiple centers (Deprez et al., 2018; Fox et al., 2012; Jovicich et al., 2014), a few previous studies that addressed test–retest reproducibility (Huang et al., 2012; Shou et al., 2013; Zhang et al., 2019) found results comparable to ours. DTI‐derived metrics are calculated from multiple images and, thus, are inherently more noisy and affected by coregistration errors and other types of stability‐related issues (Morey et al., 2010). In addition, DTI is highly prone to voxel‐level motion of the subject, which would lead to various types of intensity‐modulating artifacts (Alexander, Lee, Wu, & Field, 2006; Ni, Kavcic, Zhu, Ekholm, & Zhong, 2006). Finally, a long retest interval may introduce technical and biological effects in the test retest analysis, which may partially explain the slightly lower DTI reproducibility we found for HCP, compared to Kirby21.

Regionally, we found more variation in the DTI ICCs than in the volumetric ICCs. Again, small parcels, which are more susceptible to noise and partial volume effects (Deprez et al., 2018; Vollmar et al., 2010), and parcels in the extremes of the sample (e.g., brainstem, extreme frontal and occipital areas), where the mapping is more challenging, tended to have lower ICCs. In addition, labels with a clearly predominant direction of fibers (high FA) tended to have high ICC, which was corroborated by the observed higher reducibility for an anisotropic phantom compared to human subjects (Morey et al., 2010). This has to be taken in account when planning or interpreting the results of clinical studies. Since DTI measures experience large variability, their sensitivity to detect biological effects may be low. For instance, our power analysis revealed that, while the scan session has a very small effect size in the volumetric analysis (and thousands of subjects would be needed to detect volumetric differences), the effect size of different sessions is much higher for FA, and less than hundred subjects would be needed to detect a significant difference between scan sessions for the same individuals, in areas of low ICC. Therefore, group differences in DTI metrics must be carefully evaluated depending on effect size, location, and related technical conditions.

Despite the lower reproducibility of DTI compared to T1 volumetric data analysis, the regional ICCs were, in general, high, and the measurement variance was still lower than that of the population variance (the distance between rest–retest pairs was lower than the distance among difference subjects, as demonstrated in Figure 6), revealing that MRICloud is a reasonably stable tool.

### 5.3 Reproducibility of rsfMRI results

The I2C2s for rsfMRI data, processed with MRICloud, were lower than those for volumetric and DTI data; the averaged ICCs for the regional correlations among seeds fluctuated around 0.4. Although there are a few reports of higher ICCs, the majority of previous studies that addressed the reproducibility of rsfMRI across individuals are in agreement with our findings (Andellini, Cannata, Gazzellini, Bernardi, & Napolitano, 2015; Deprez et al., 2018; Huang et al., 2012; Shou et al., 2013). As for the DTI data, but on larger scale, the (well‐known) rsfMRI low reproducibility is attributed not only to the postprocessing (which is extremely variable in methodology), but also to the actual nature of the sequence (Birn et al., 2013; Noble et al., 2017; Patriat et al., 2013). For instance, here we found reproducibility slightly superior for HCP data than for Kirby21. HCP has more frames per run and higher resolution that Kirby21, which may have contributed to the observed difference. Multiple technical factors (magnetic fields, sequence artifacts, motion) and biological conditions (physical and mental states, even in healthy individuals) contribute to data variability, some with a biologically relevant effect, and others as just noise (Airan et al., 2016; Kelly, Biswal, Craddock, Castellanos, & Milham, 2012). To isolate and quantify the contribution of each of these factors is one of the biggest challenges in the field and is not within the scope of this study.

We found that the reproducibility of the outputs of MRICloud is comparable, and slightly higher, to that obtained using SPM CONN. As the parcellation scheme applied in both methods is the same, as well as most of the postprocessing steps (slice‐time correction, coregistration, motion correction, outlier rejection, nuisance correction, etc.), the differences in the reproducibility are likely attributable to methodological differences in the image mapping. Likewise in the T1‐volumetric analysis, while this seems to have low influence in anatomical normal data, the differences in mapping may affect anatomically abnormal data differently and the indices of reliability may present a great variation, both absolutely and comparatively, among methods.

## 6 CONCLUSION

We tested–retested the reproducibility of MRICloud, a free, automated method for multimodal MRI segmentation and quantification, on two public, independent datasets. The reproducibility was extremely high for T1‐volumetric analysis, high for DTI (however, regionally variable), and low for resting‐state fMRI. The reproducibility for T1‐volumetric analysis and rsfMRI slightly over performed that of widely used software. The knowledge about the global reproducibility of each modality pipeline, as well as the regional reproducibility for each label, is essential for both study planning and data interpretation and is in line with the efforts to increase reproducibility and transparence in science.

## CONFLICT OF INTEREST

None declared.

## Data Availability

The data analyzed in this study are publicly available as Tables [Link], [Link], [Link].
